# Downstream Products are Potent Inhibitors of the Heparan Sulfate 2-O-Sulfotransferase

**DOI:** 10.1038/s41598-018-29602-4

**Published:** 2018-08-07

**Authors:** David F. Thieker, Yongmei Xu, Digantkumar Chapla, Chelsea Nora, Hong Qiu, Thomas Felix, Lianchun Wang, Kelley W. Moremen, Jian Liu, Jeffrey D. Esko, Robert J. Woods

**Affiliations:** 1 Department of Biochemistry and Molecular Biology, University of Georgia, Athens, GA 30602 USA; 2 Complex Carbohydrate Research Center, University of Georgia, Athens, GA 30602 USA; 3 Division of Chemical Biology and Medicinal Chemistry, Eshelman School of Pharmacy, University of North Carolina, Rm 1044, Genetic Medicine Building, Chapel Hill, USA; 4 Department of Cellular and Molecular Medicine, University of California San Diego, La Jolla, California USA

**Keywords:** Glycobiology, Computational models, Transferases, Thermodynamics, Polysaccharides

## Abstract

Heparan Sulfate (HS) is a cell signaling molecule linked to pathological processes ranging from cancer to viral entry, yet fundamental aspects of its biosynthesis remain incompletely understood. Here, the binding preferences of the uronyl 2-*O*-sulfotransferase (HS2ST) are examined with variably-sulfated hexasaccharides. Surprisingly, heavily sulfated oligosaccharides formed by later-acting sulfotransferases bind more tightly to HS2ST than those corresponding to its natural substrate or product. Inhibition assays also indicate that the IC_50_ values correlate simply with degree of oligosaccharide sulfation. Structural analysis predicts a mode of inhibition in which 6-*O*-sulfate groups located on glucosamine residues present in highly-sulfated oligosaccharides occupy the canonical binding site of the nucleotide cofactor. The unexpected finding that oligosaccharides associated with later stages in HS biosynthesis inhibit HS2ST indicates that the enzyme must be separated temporally and/or spatially from downstream products during biosynthesis *in vivo*, and highlights a challenge for the enzymatic synthesis of lengthy HS chains *in vitro*.

## Introduction

Glycosaminoglycans (GAGs) are a group of linear polysaccharides that consist of alternating hexosamine and hexuronic acid units. With the exception of hyaluronan, GAGs are variably sulfated, and are categorized into heparan sulfate (HS), chondroitin sulfate, and dermatan sulfate based on the composition and linkage of the monosaccharide residues^1^. HS has received considerable attention due to the broad functionality it displays in key cellular signaling events that are crucial for proper development^2^, such as vasculogenesis^3^, axon guidance^4^, and cellular differentiation^5^. The critical role for HS within animals, as well as its presence on cell surfaces, has provided pathogenic organisms with an accessible route for attachment and entry into the cell. Families of viruses including *Herpesviridae*^6^, *Flaviviridae*^7^, and *Filoviridae*^8^ each exploit HS for cell adhesion. HS has also been implicated in various forms of cancer^9^ and may either promote or inhibit metastasis depending on the sulfation pattern along the carbohydrate chain^10^. Efforts to characterize HS-protein interactions indicate that some binding modes recognize specific sulfation patterns, while others are non-specific and appear to be dependent primarily on negative charge density^11,12^. The prototypical example of highly specific HS binding is that between antithrombin and heparin (a more highly sulfated form of HS bearing antithrombin binding sites) whose affinity increases more than 1000-fold upon 3-*O*-sulfation of a key hexosamine residue^13–16^. There is growing interest in the therapeutic potential of HS mimetics due to the diverse functionality and possibility for highly specific interactions^17–19^.

**Figure 1:**
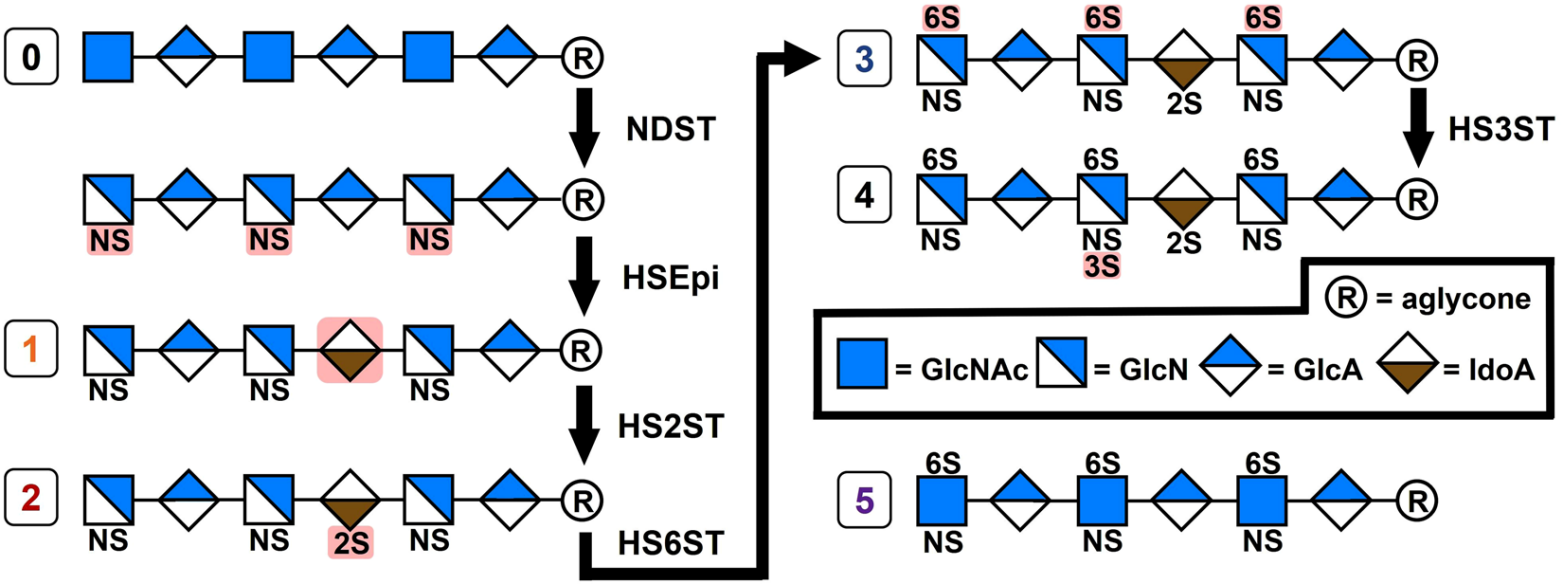
Heparan Sulfate (HS) biosynthetic pathway. After formation of the linkage region and polymerization of the chain (not shown), the chains undergo five modification steps catalyzed by corresponding enzymes, including the GlcNAc N-Deacetylase-N-Sulfotransferase (NDST), GlcA C5 Epimerase (HSEpi), 2-O-Sulfotransferase (HS2ST), 6-O-Sulfotransferase (HS6ST), and 3-O-Sulfotransferase (HS3ST). Differences from the products of the individual enzymes are highlighted in red. Five hexasaccharides were synthesized for this study and are numbered accordingly.

Biosynthesis of HS begins with the assembly of a core tetrasaccharide to specific serine residues of proteins^20^. Two enzymes then add alternating *N*-acetyl glucosamine (GlcNAc) and glucuronic acid (GlcA) residues until the chain reaches between 20 and 150 disaccharide units in length^21^. Assembly of the HS chain continues via a set of enzymes that modify the repeating disaccharide unit, including concomitant *N*-deacetylation and *N*-sulfation of GlcNAc units^22^, epimerization of GlcA to iduronic acid (IdoA), 2-*O*-sulfation of uronic acids and 6-*O-* and 3-*O*-sulfation of hexosamine units (Fig. 1). A sequential biosynthetic pathway was originally proposed based on label-chase studies of HS in mouse mastocytoma microsomal fractions^23^. In general, the enzymatic preferences for upstream substrates and the inactivity of downstream products for each enzyme support a sequential pathway^24^, but cannot explain the irregular sulfation patterns observed *in vivo*. A method of regulating enzyme-substrate interactions has been proposed to resolve this discrepancy, in which the HS biosynthetic enzymes form a macro-complex termed the GAGosome^1,20^. Although a GAGosome involving all of the biosynthetic enzymes has not been detected directly, the epimerase and 2-*O*-Sulfotransferase (HS2ST) interact *in vitro*^25^ and co-localize in the medial Golgi *in vivo*^26^.

**Figure 2:**
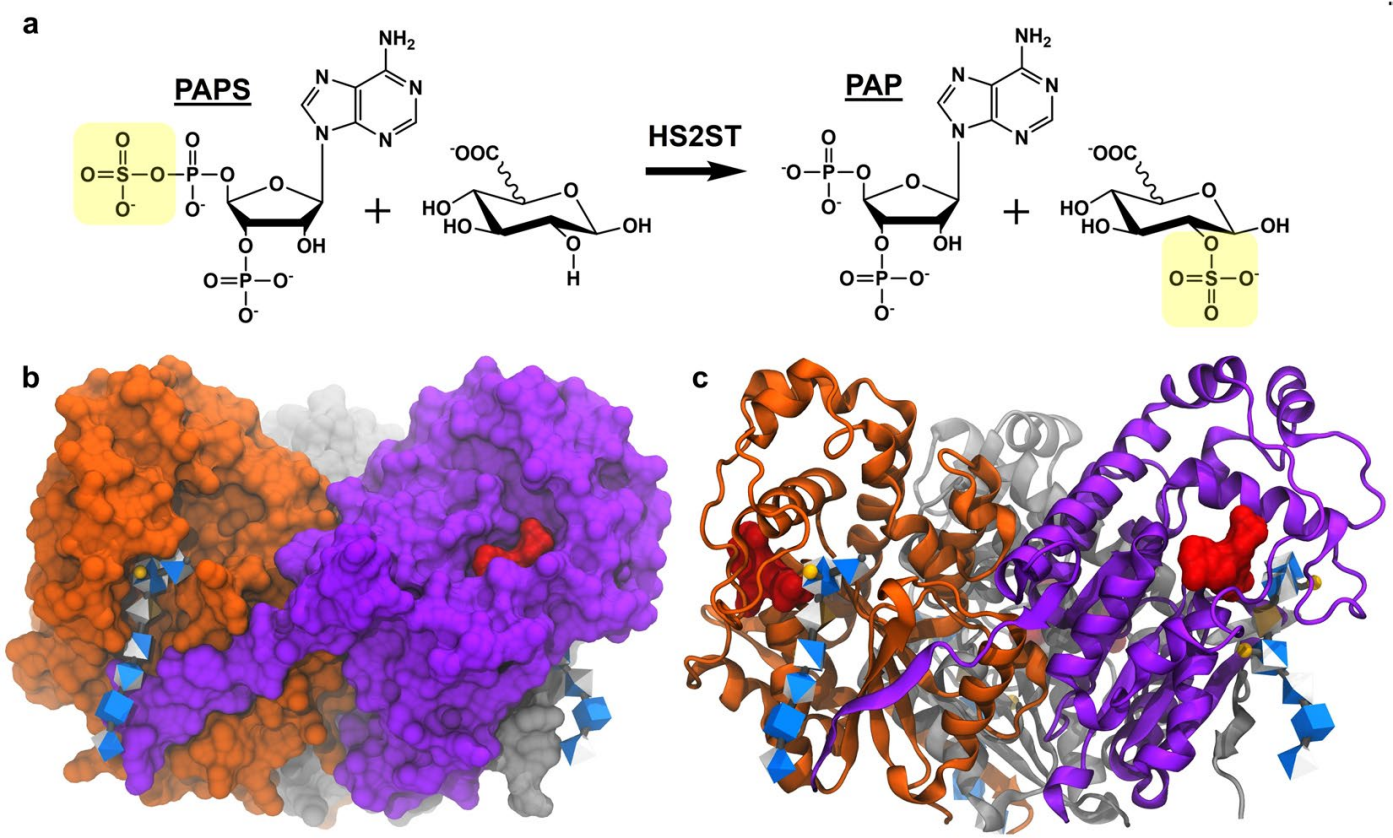
Structure of the 2-O-Sulfotransferase (HS2ST). **(a)** Reaction scheme for the HS2ST enzyme. The sulfate (yellow) from PAPS is transferred to the 2-position of a uronic acid (UA), resulting in PAP as a byproduct. **(b)** The co-crystal of the trimeric HS2ST in complex with PAP (red) and a HS heptasaccharide (3D-Symbol Nomenclature for Glycans^76^), sulfate depicted with yellow spheres). **(c)** The protein is depicted in a ribbon structure to convey the three symmetrical active sites that contain both PAP and HS residues.

Only one isoform of the HS2ST enzyme has been identified^27^, in contrast to the multiple isoforms with varying specificities that exist for all other HS sulfotransferases^20^. Bullock *et al*.^28^ have shown that HS2ST is critical for normal development in mice. HS2ST catalyzes the transfer of a sulfate moiety from the co-factor 3′-phosphoadenosine-5′-phosphosulfate (PAPS) to the second hydroxyl position (2-sulfation) of a uronic acid (UA) residue (Fig. 2a); a motif that is required for HS recognition by proteins such as fibroblast growth factor-2^29^. Although HS2ST is capable of transferring sulfate to either GlcA or IdoA residues *in vitro*, GlcA2S residues are sparse *in vivo* in contrast to IdoA2S^27,30^. The scarcity of GlcA2S may be due to co-localization of the epimerase and the HS2ST enzymes^26^, and the preference of the enzyme for IdoA (2.5-fold enhancement towards IdoA)^27^. The HS2ST enzyme exists as a homotrimeric complex, in which the C-terminus of one monomer extends to the substrate binding site in the neighboring monomer^27^ (Fig. 2b,c). Although -IdoA2S-GlcNAc- units were detected within HS and heparin samples^31,32^, the enzyme strictly required a -GlcNS-UA-GlcNS- motif for catalysis *in vitro*^33^. Absence of either *N*-sulfate (NS) abolished activity, as did the presence of a sulfate at the 6-position (6S) on the reducing side glucosamine unit. Additionally, the minimum oligosaccharide size for activity was determined to be a pentasaccharide.

A lack of activity for HS2ST with 6-*O*-sulfated substrates is consistent with a sequential mechanism of assembly of the chain; therefore, little attention has been paid to the affinity or inhibitory ability of downstream biosynthetic products. Here, we present equilibrium binding data for hexasaccharides with varying sulfation patterns that correspond to steps within the HS biosynthetic pathway (Fig. 1 and Supplementary Table 1). Unexpectedly, binding and enzyme activity inhibition assays led to the observation that downstream products of HS biosynthesis are effective inhibitors and bind with high affinity to HS2ST. Biding studies confirmed the enhanced binding of recombinant HS2ST in the biological context of full length HS as well. These observations raised several questions, including why downstream products do not inhibit the enzyme *in vivo*, and by what mechanism they bind to and inhibit HS2ST. We explored the latter question through targeted point mutagenesis, as well as docking and molecular dynamics (MD) simulations. All downstream oligosaccharides were predicted to be able to fit into the HS2ST active site and remained bound to the protein over the course of the 200 ns MD simulations. Moreover, the inactivity of the 6-*O*-sulfated oligosaccharides as substrates was predicted to arise from the positioning of the 6-*O*-sulfate in the PAPS sulfate-binding site, presumably preventing the co-factor from binding.

## Results

### Binding analyses indicate sulfation-dependent HS2ST binding preferences

HS-protein binding studies often utilize biologically-derived oligosaccharides of heterogeneous length and sulfation^34,35^; however, the synthesis of oligosaccharides with defined length and specific sulfation patterns has recently become possible^36^. We employed biolayer interferometry (BLI) to determine equilibrium surface binding constants (K_D_) for HS2ST with synthetic HS hexasaccharides (Fig. 1 and Supplementary Table 1). The HS compounds were attached to the BLI biosensor surface to conserve sample and to ensure a significant signal upon protein binding. The HS compounds contained a biotinylated PEG4 linker at the reducing terminus, enhancing the likelihood of a consistent presentation of the linear glycan on the sensor surface, in contrast to immobilizations via intra-chain modifications^37^.

**Figure 3:**
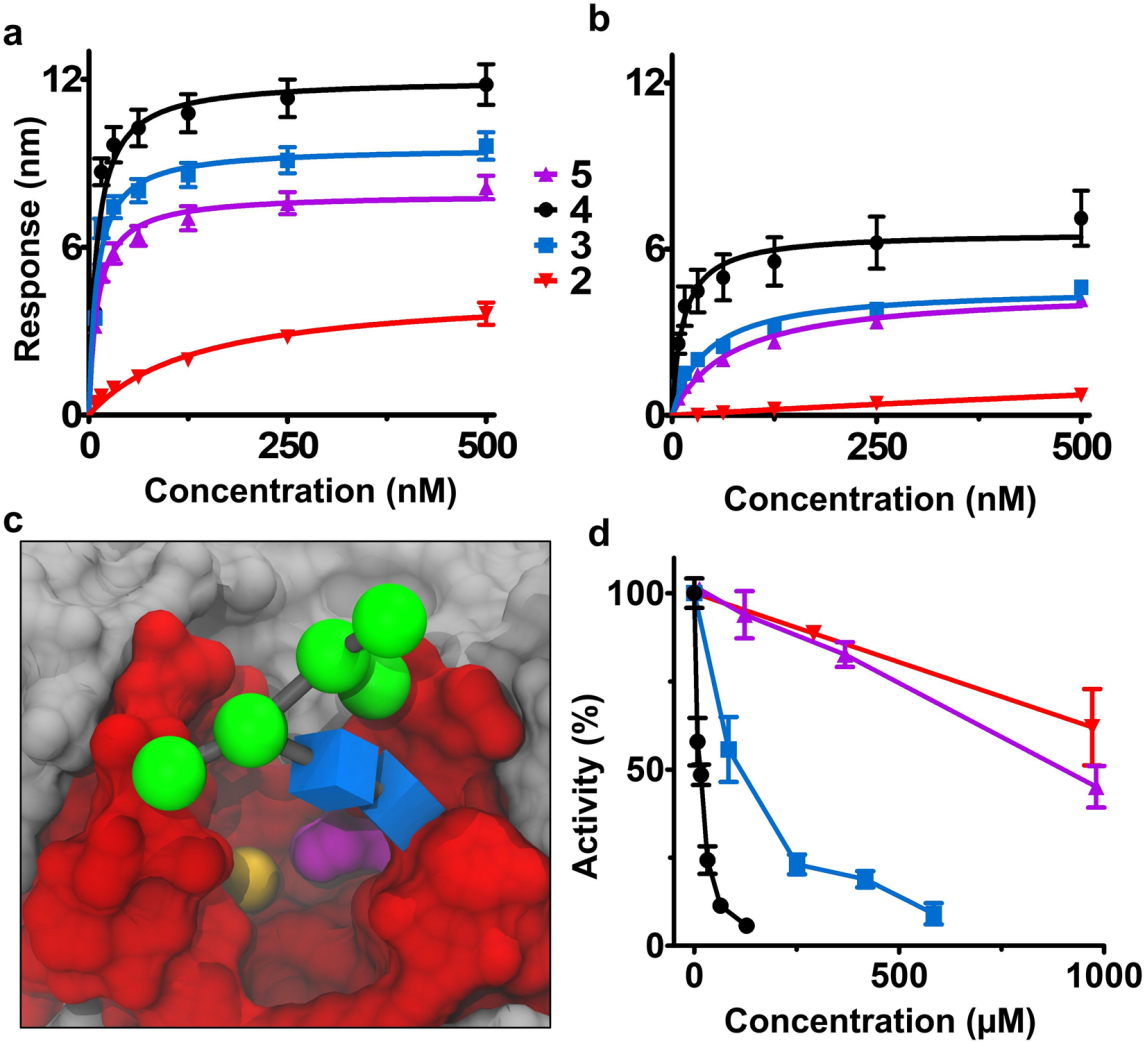
HS2ST WT binding and inhibition assays. **(a)** Equilibrium binding curves (n = 3) for glyc-HS2ST with compounds **2, 3, 4**, and **5** as red, blue, black, and purple, respectively. **(b)** Equilibrium binding curves (n = 3) for non-glyc-HS2ST **(c)** A model of glyc-HS2ST was created by adding a short, high-mannose glycan (green and blue) to N108 of the crystal structure (PDB ID: 4NDZ). The trimeric protein is depicted in gray, except for amino acids within 10 Å of the acceptor IdoA residue (red) and the catalytic H142 (magenta). The modeled sulfate from PAPS is also depicted as a yellow sphere. **(d)** Inhibition assays in which a single concentration of non-glyc-HS2ST and substrate was incubated with varying concentrations of HS hexasaccharides. Errors are reported as the standard deviations of the mean.

Binding constants were collected for two isoforms of the enzyme; a glycosylated human variant (glyc-HS2ST) expressed in mammalian cell lines, and a highly homologous (96% identity) non-glycosylated chicken isoform expressed in *E*. *coli* (non-glyc-HS2ST). Glyc-HS2ST demonstrated modestly enhanced binding responses (Fig. 3a,b) and lower K_D_ values (Table 1) than the non-glyc-HS2ST construct. The high sequence identity suggested that the differences in binding primarily resulted from glycosylation of the enzyme. Glycosylation generally dampens protein dynamics^38–40^ and has been shown to impair enzyme activity^38,40^. Thus, the enhanced ligand affinity seen in HS2ST was unexpected and may relate to the specific position of the glycosylation site (N108)^41^. This site is proximal to the HS binding site (Fig. 3c), and MD simulations suggest the glycan may stabilize HS binding by capping the substrate in the binding site.

**Table 1:** Equilibrium K_D_ values (nM)^a^ for the interaction of HS2ST constructs with varying HS compounds, as calculated from saturation binding curves obtained with BLI.

Compound	WT	WT	H142A	H142K	H142R	H142E	R189A
	glyc-HS2ST	non-glyc-HS2ST					
2	138 ± 19^b^	—^c^	460 ± 91	198 ± 34	136 ± 20	—	—
3	10 ± 1	42 ± 5	20 ± 3	15 ± 2	12 ± 1	41 ± 4	448 ± 124
4	10 ± 2	13 ± 3	10 ± 2	11 ± 2	10 ± 1	19 ± 3	63 ± 5
5	11 ± 1	72 ± 8	25 ± 2	22 ± 2	13 ± 1	140 ± 11	—

^a^n = 3.

^b^Standard errors of best fit parameters determined by nonlinear regression.

^c^Response too weak to fit.

Interestingly, the affinity of the hexasaccharide that resembles the natural substrate of the reaction (**1**) was too weak to measure for either HS2ST isoform, but both displayed measurable affinities for sulfated molecules despite the lack of enzyme activity associated with these compounds (Table 1). The non-glyc-HS2ST demonstrated a clear trend in which the affinity increased according to the degree of HS sulfation. High affinities were observed for 6-*O*-sulfated compounds (**5**, **3**, and **4**) with glyc-HS2ST, but the similarity of their values (Table 1) likely reflects the limit of the sensitivity of the BLI assay (7.8 nM was the lowest concentration of protein whose binding was detectable). Since glycosylation of the enzyme did not appear to affect the binding preferences of the enzyme, and bacterial expression was more convenient, the remainder of the study utilized non-glyc-HS2ST.

### HS2ST binding preferences reflect inhibitory potential

The tight binding of the higher-sulfated compounds suggested that they might also act as inhibitors of the HS2ST enzyme. Inhibition assays were thus performed with non-glyc-HS2ST and all compounds except for **1**, the enzymatic substrate. As in the case of the affinities, the IC_50_ values were found to depend strongly upon the degree of sulfation of the hexasaccharide (Fig. 3d and Table 2). Compound **5** appeared to inhibit at least as well as the product of the reaction (**2**); however, the IC_50_ was not reached even at the maximum substrate concentration (1 mM) for either **2** or **5**. The addition of a single sulfate moiety enhanced the potency of the glycan as an inhibitor by ~10-fold (compare **3** and **4**). The presence of both 2- and 3-O-sulfate moieties in the hexasaccharide (**4**) enhanced its inhibitory potential by approximately 100-fold relative to **5**. These *in vitro* inhibition assays indicate that downstream products of the HS biosynthetic pathway could interfere with HS2ST catalysis *in vivo*.

**Table 2:** IC_50_ values (nM) of the various HS hexasaccharides, as determined from inhibition assays of the reaction between HS2ST and completely de-*O*-sulfated *N*-sulfated heparin.

Compound	non-glyc-HS2ST
2	>1000^a^
3	14^b^
4	114^b^
5	>1000^a^

^a^n = 2.

^b^n = 3.

### Downstream HS inhibitors bind within the HS2ST active site

**Figure 4:**
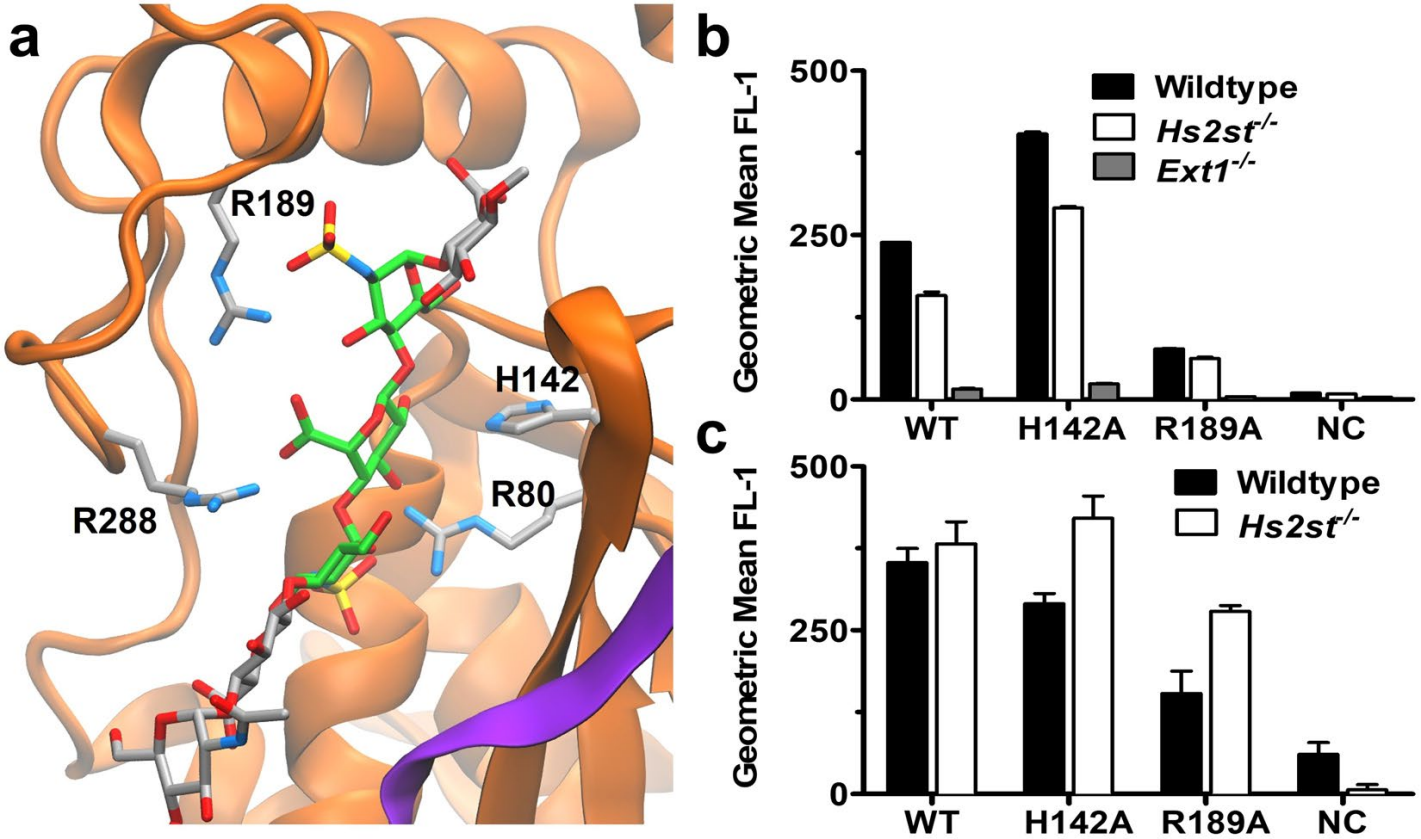
HS2ST active site mutations. **(a)** The crystal structure of the co-complex (PDB ID: 4NDZ) is depicted without PAP, for clarity. HS2ST monomers are distinguished by orange and purple ribbons, and mutated amino acids side-chains are depicted in gray. The glycan is depicted in gray, except for the acceptor (IdoA) and flanking GlcNS residues, which are highlighted in green. **(b)** Flow cytometry of non-glyc-HS2ST constructs with various CHO cell lines (n = 2). **(c)** Flow cytometry of non-glyc-HS2ST constructs with various MLE cell lines (n = 3). Errors are reported as the standard deviations of the mean.

To assess whether the inhibitory compounds interact with the active site, point mutations were introduced in non-glyc-HS2ST (Fig. 4a). Four mutants of the catalytic residue (H142A/E/R/K) were created, along with a mutation of the residue that facilitates 2-*O*-sulfation of IdoA residues (R189A)^27^. Additionally, a negative control (NC) was generated containing multiple mutations of positively charged residues (R189A, R80E, and R184E) that reduced the net charge of each protein monomer by 5. The extent that the mutations affected affinity provided an indication that the ligand had been bound in the active site (Table 1 and Supplementary Fig. 1). While R189A markedly diminished the affinity for all compounds, the NC mutations reduced affinity to immeasurable levels. Removal of the catalytic side chain (H142A) enhanced the affinity of each compound, compared to the WT, by approximately 2–3-fold. Addition of a positive charge at this location (H142K/R) further enhanced the affinity for each compound, so much so that a K_D_ value could be measured even for **2**. The H142E mutation diminished the affinity of **5** by half compared to WT, but its effects were less significant for binding to **3** or **4**. The basic character of the catalytic residue (H142) in the WT enzyme may be significant enough to be approximately comparable to that of a glutamate in this specific microenvironment.

### HS2ST mutants display differential binding to HS-producing cells

The interaction of WT HS2ST and mutants with biologically relevant full-length HS was examined using flow cytometry with binding being detected by fluorescently-labeled antibody to the maltose binding protein tag on the recombinant HS2ST. Chinese hamster ovary (CHO) cells lack the HS3ST enzyme^42^; therefore, the cell surface HS chains do not contain compound **4**. Non-glyc-HS2ST constructs were incubated with wild-type CHO cells, as well as with CHO cells bearing mutations in different biosynthetic enzymes (Fig. 4b). Cell-surface binding reproduced the general trends in affinities observed with synthetic oligosaccharides. Specifically, the H142A mutant showed the highest affinity for Wildtype CHO cells, and diminished in affinity in the order of WT, R189A, and NC. The HS produced by the *Hs*2*st*^−/−^ cells has no detectable 2-*O*-sulfate groups, and this loss is accompanied by an increase in the amount of both *N*- and 6-*O*-sulfate groups^43^. Thus, the composition of HS produced by *Hs2st*^−/−^ is increased for **1** and **5** and decreased for **2** and **3** compared to Wildtype cells. Since the HS2ST constructs demonstrated a tighter affinity for **2** and **3** compared to **1** and **5** in the previous BLI studies, the lower binding signal for the *Hs2st*^−/−^ cells is understandable. Negligible binding for each of the HS2ST constructs was observed in *Ext1*^−/−^ cells, which lack HS. This finding suggests that the HS2ST does not interact with other glycosaminoglycans, such as chondroitin sulfate, which are up-regulated in this cell line^44^. Collectively, the cell binding data are consistent with the oligosaccharide binding assays and reinforce the hypothesis that the products of the early-acting HS sulfotransferases must be temporally and/or spatially separated from the products of the later enzymes in the biosynthetic pathway.

A separate flow cytometry assay was conducted with Wildtype and *Hs2st*^−/−^ mouse lung endothelial (MLE) cells (Fig. 4c), which produce HS3ST. The *Hs2st*^−/−^ MLE cells demonstrated a similar trend in affinities towards the HS2ST constructs as was found with the CHO cells and BLI experiments; namely, enhancing affinity in the order of NC, R189A, WT, and H142A. Interestingly, HS2ST constructs demonstrated enhanced binding with the *Hs2st*^−/−^ MLE cells compared to Wildtype. This difference could suggest a role for 3-*O*-sulfate in HS2ST binding; however, we were unable to directly compare the relative contributions of each moiety to HS2ST binding as our hexasaccharide library did not include a compound that both lacked 2-*O*-sulfate and included 3-*O*-sulfate. Alternatively, enhanced binding of *Hs2st*^−/−^ compared to WT cells may result from a difference in total charge of the HS chains on the cell surface.

### Predicted structures of HS2ST-inhibitor complexes

Docking and MD simulations were performed to structurally characterize the binding mechanism of HS2ST with each of the hexasaccharides. The non-glyc-HS2ST enzyme has been crystallized in complex with PAP, a byproduct of the reaction, and a heptasaccharide substrate (GlcA-GlcNAc-GlcA-GlcNS-IdoA-GlcNS-GlcA-PNP) that is nearly identical to **1** (GlcNS-GlcA-GlcNS-IdoA-GlcNS-GlcA-PNP), except for the presence of a non-reducing terminal GlcA-GlcNAc unit in the former (PDB ID 4NDZ^33^). The hexasaccharides were docked to unliganded (apo) HS2ST using Vina-Carb^45^. As a positive control, the equivalent hexasaccharide (lacking the non-reducing terminal GlcA) was created with GLYCAM-Web (www.glycam.org/gag) and docked into the active site of HS2ST, with the same level of flexibility as employed for the other compounds. The pose of the top-ranked model matched that of the ligand in the co-crystal (RMSD of 1.0 Å) (Supplementary Fig. 2a), validating the docking protocol. The remaining compounds were created similarly and docked into the HS2ST active site.

**Figure 5:**
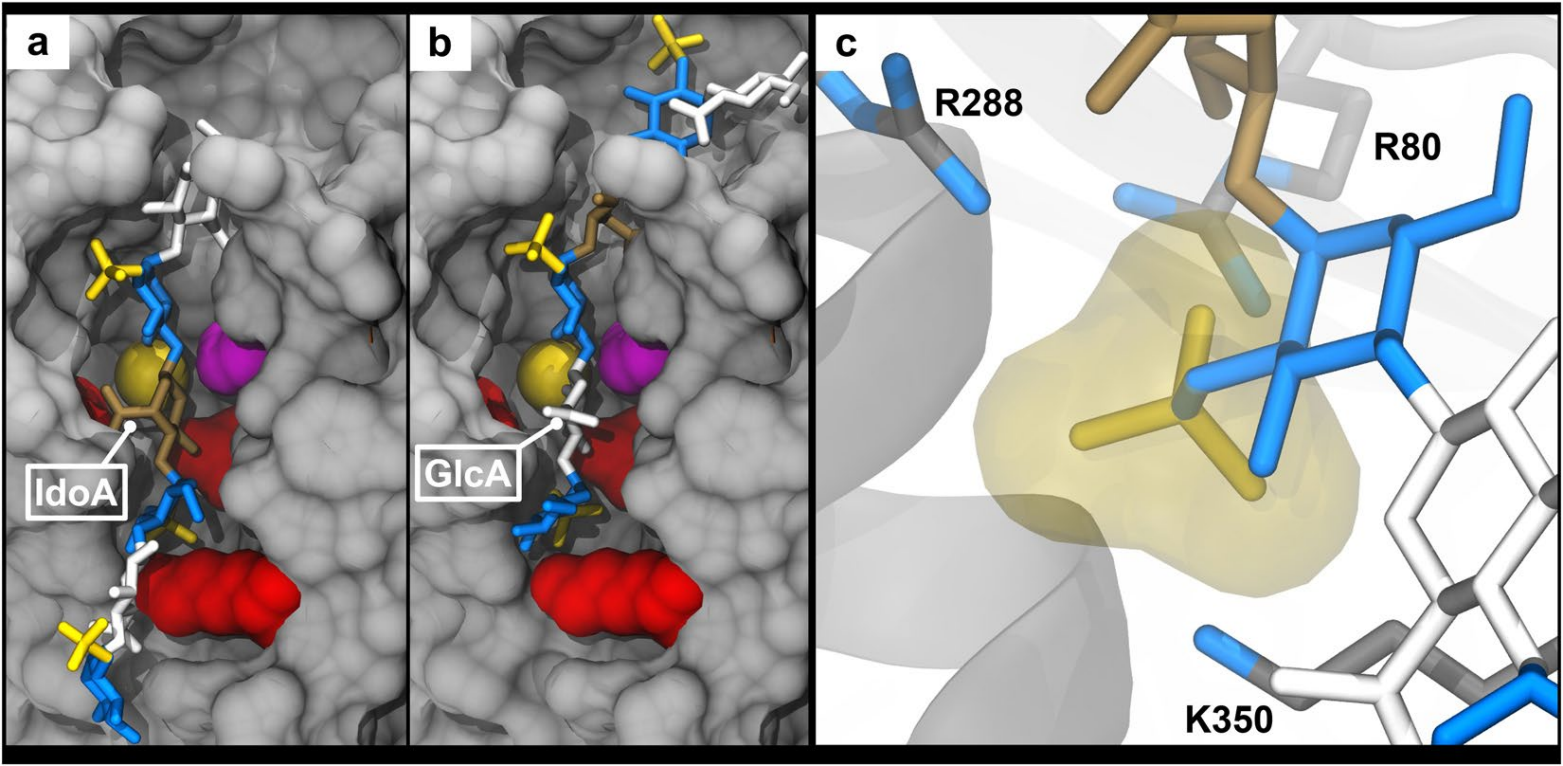
Predicted structures of HS2ST with compound 1. The same glycan color scheme is used as Fig. 4. **(a,b)** Amino acids that form the *N*-sulfate binding pocket (R288, R80, and K350) are colored red and the catalytic H142 is magenta. The sulfur atom modeled onto PAP is displayed for context (yellow sphere). **(a)** Docked model of **1** that matches the crystal structure. **(b)** Docked model of **1** that is translocated one disaccharide unit, resulting in GlcA as the acceptor residue. **(c)** A close-up view of the *N*-sulfate binding pocket from the crystal structure (PDB ID: 4NDZ) with the three amino acids coordinating the *N*-sulfate. The transparent, yellow surface represents the VDW radii of the sulfate moiety.

Results from the docking of **1** indicated two potential modes of binding (Fig. 5). In the top ranked pose, the entire glycan was translocated one disaccharide unit within the binding groove, into a position where a GlcA was poised to receive the sulfate from PAPS, rather than IdoA. The second binding mode matched that seen in the co-crystal structure (RMSD of 1.1 Å). Translocation of the oligosaccharide appeared to be promoted by the formation of electrostatic interactions between the *N*-sulfate and three positively charged protein residues (R288, R80, and K350). Indeed, these same amino acids coordinate with the sulfate moiety of the GlcNS that flanks the acceptor residue on the non-reducing side in the co-crystal structure (Fig. 5c). We refer to this site as the *N*-sulfate binding pocket. Notably, flanking GlcNS residues are required for HS2ST activity33; a feature that is now explained in terms of the enhanced stability provided by the interactions between the NS sulfate and R288/R80/K350.

**Figure 6:**
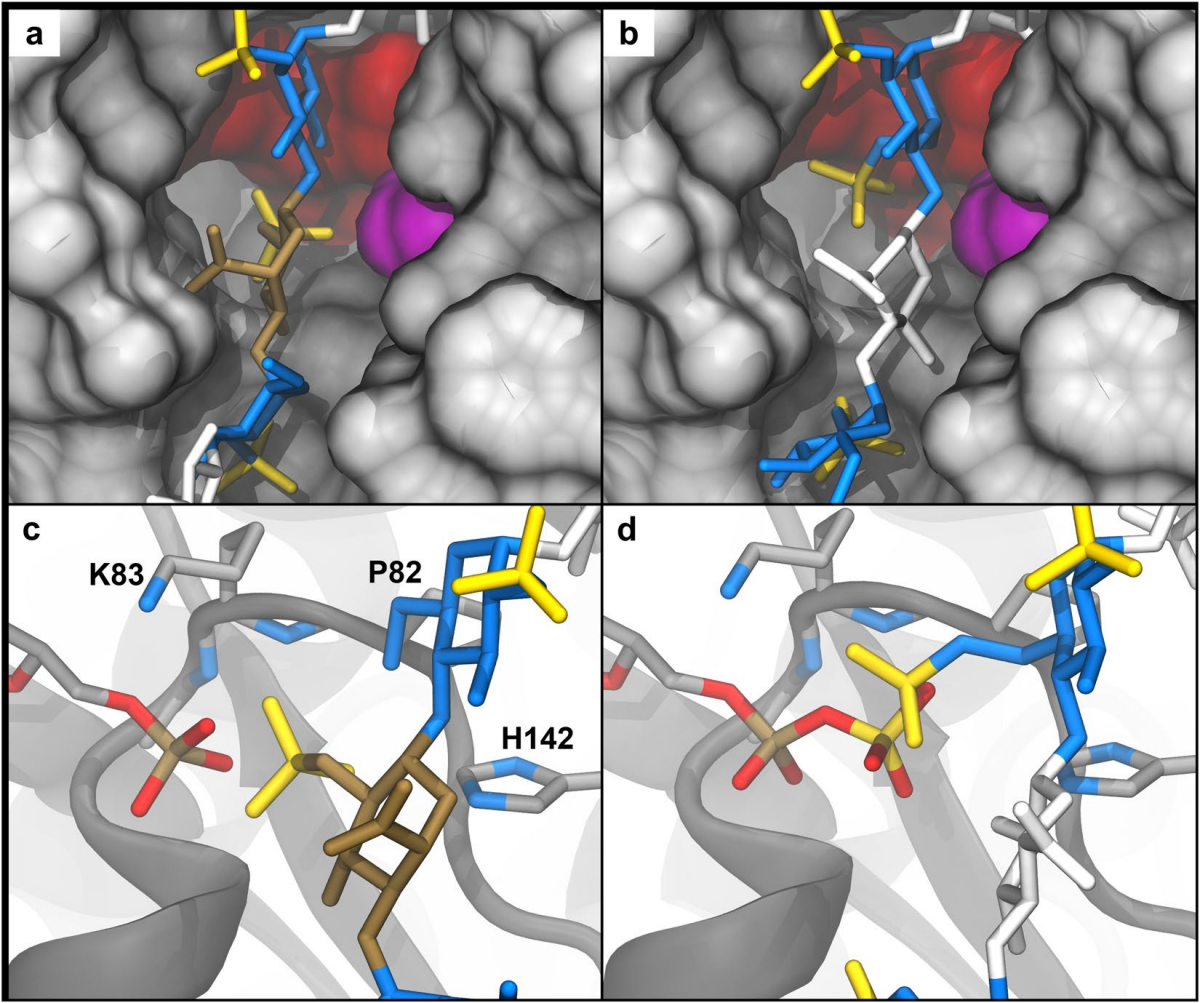
Predicted structures of HS2ST with non-substrate HS sequences that occupy the Phospho-Sulfate Binding (PSB) loop. (**a,c**) Docked model of compound 2. (**b,d**) Docked model of compound 5. (**a,b**) The trimeric protein is depicted in gray, except for the catalytic residue (H142A; magenta) and the PSB loop (T84, K83, and P82; red). (**c,d**) The catalytic residue and residues from the PSB loop are depicted in dark gray. Residues 180–191 are hidden for clarity. PAP or PAPS is displayed for context (gray, left), but was not present during the docking procedure.

The top-ranked model from docking the product of the reaction (**2**) was in essentially the same position as was found in the co-crystal of HS2ST with substrate (Fig. 6a,c). The 2S moiety was positioned where the sulfate from PAPS would reside, in the canonical phospho-sulfate binding (PSB) site that is shared amongst all sulfotransferases^46^. This pocket is favorable for a sulfate due to interactions with backbone amides (T84 and K83) that are oriented towards the PAPS phospho-sulfate moiety, along with the side chain of K83. Although **5** was not expected to bind due to a previously predicted steric clash between its 6-*O*-sulfate and P82/Y173^33^, the top-ranked model swiveled the 6-*O*-sulfate into the PSB site (Fig. 6b,d) enabling **5** to bind. The model was also translocated by one disaccharide unit, similar to the result for **1**. In the case of **5**, the 6-*O*-sulfate adopted a position equivalent to that occupied by the 2-*O*-sulfate in the modeled complex of **2**. Results from **3** indicate that compounds containing both 2S and 6S moieties are capable of binding in a similar orientation as in **2** (Supplementary Fig. 2b). Interestingly, addition of a 3S moiety to the non-reducing flanking GlcNS residue (**4**) appeared to prevent that residue from positioning the *N*-sulfate moiety in the *N*-sulfate binding pocket, resulting in a top-ranked model that was again translocated by one disaccharide unit (Supplementary Fig. 2c,d).

Docking also generated poses in which the oligosaccharide was placed in a reversed orientation (flipped end-for-end) relative to the co-crystal structure (Supplementary Fig. 2e). This binding mode only occurred with 2-sulfated compounds (**2, 3, 4**), placing the 2S in the *N*-sulfate binding pocket and the NS in the PSB pocket. While plausible for binding, these modes are not appropriate for catalytic activity.

A 200 ns MD simulation of the trimeric apo-protein indicated an unexpected mobility of the protein loops proximal to the active site (Supplementary Fig. 3). By the end of the simulation, each of the three active sites of the trimer adopted topologically distinct conformations; one domain maintained a conformation similar to the crystal structure, another opened to expose the PAPS and substrate-binding sites, while the third closed over the substrate-binding site. A simulation of the non-sulfated HS-precurser (**0**) bound to HS2ST was performed to determine whether the complex was stable, given that HS2ST does not act on this oligosaccharide. Simulations of each of the other compounds were also performed, including a translocated model of **1**. All ligands remained bound during the simulation, although in many instances the active site loops showed motions as seen in the apo-HS2ST simulations. The ligand mobility (RMSF) and interaction energy for each compound was collected to determine the stability of the complexes (Table 3). HS precursor **0** demonstrated significantly enhanced positional fluctuations than the sulfated compounds, as well as showed the weakest interaction energy. Surprisingly, both starting positions of **1** appeared equally stable during the trajectory. The interaction energies of HS compounds largely corresponded to the experimentally observed trends in affinities, except for compound **5**, whose binding energy was predicted to be lower than that of **2**; possibly suggesting that the bound orientation of **5** differs from the pose selected from docking.

**Table 3:** Root mean square fluctuations (RMSF) and interaction energies (ΔG_bind_)^a^ obtained from MD simulations of models of compounds 0–5 docked into the HS2ST active site.

	RMSF (Å)	ΔG_bind_ (kcal/mol)
0 Aligned	4.6 ± 3.2^b^	−169 ± 11^b^
1 Aligned	1.4 ± 0.5	−263 ± 11
1 Translocated	1.4 ± 0.7	−268 ± 10
2 Aligned	1.0 ± 0.5	−327 ± 12
3 Aligned	1.2 ± 0.6	−343 ± 13
4 Translocated	1.2 ± 0.6	−375 ± 14
5 Translocated	1.6 ± 1.0	−310 ± 13

Docked models are described as either in-register (Aligned) with regards to the structure of hexasaccharide-bound HS2ST (PDB ID: 4NDZ), or out-of-register (Translocated).

^a^Values were calculated for the latter 50 ns of the 200 ns trajectory and do not include entropy contributions.

^b^Standard deviation.

## Discussion

This study of the HS2ST binding specificity presents the surprising discovery that downstream HS products demonstrate higher affinity than both the enzymatic substrate and product of the HS2ST reaction, presenting an interesting challenge for biosynthesis. Considering the efficiency of *2*-O-sulfation *in vivo*^47^, a mechanism for separating HS2ST from fully processed HS chains must exist within the cell. Chase-sulfation experiments of microsomal fractions indicated a stepwise process in which a limited amount of substrate was successively modified until the entire pool of oligosaccharides was consumed^47^, and a supramolecular complex of each of the sulfotransferases (GAGosome) was proposed as a mechanism for coupling the enzymatic reactions^23^. However, this model requires a regulatory process to maintain a unidirectional pathway and thereby avoid inhibition by downstream products. We propose a system in which small complexes of HS biosynthetic enzymes are separated into distinct Golgi stacks. This model is supported by previous studies that indicate epimerase localization is dependent on HS2ST expression^26^, whereas HS6ST isoforms localize independently of HS2ST^48^. Although discrete localization within the Golgi has not been reported for HS sulfotransferases, the functionally similar GlcNAc6ST family of enzymes is distributed in an isoform-specific manner^49^. Additional studies will be necessary to determine the cellular mechanism for regulating exposure of HS2ST to downstream products.

It was previously shown that HS2ST was inactive against substrates if glucosamine residues flanking uronic acid were 6-*O*-sulfated, and it was hypothesized that steric collisions involving the 6-*O*-sulfate groups prevented these sequences from binding in the active site of HS2ST^33^. Our results indicate that oligosaccharides containing the 6-*O*-sulfate motif bind stably in the active site, and molecular modeling suggests a mechanism in which the 6-O-sulfate moiety blocks co-factor binding by occupying the PAPS binding site, thereby preventing catalysis. The PSB pocket is a fundamental feature of sulfotransferases, and is structurally similar to the P-loop motif associated with nucleotide-binding proteins^50^. Heparin inhibition of members of the kinase family has been demonstrated previously^51,52^; however, Hathway, *et al*. also identified kinases that were unaffected by the highly sulfated GAG. Therefore, while this nucleotide-binding motif may be a general binding site for sulfate moieties, it is not sufficient for heparin inhibition.

The slightly higher affinity for product than substrate may simply be due to electrostatic interactions between the 2-*O*-sulfate and PSB loop. Although PAPS was not included in the binding assays, the cofactor was present during the inhibition assays and appeared unable to outcompete the HS fragments. An inability to release the product of the reaction would impede overall activity, so we propose a mechanism for destabilizing the product following 2-*O*-sulfation that is based upon known conformational preferences of the IdoA ring. The co-crystal structure contains an IdoA unit in the ^4^C_1_ conformation, which is unusual as this ring typically demonstrates a strong preference for the ^2^S_o_ or ^1^C_4_ conformations in solution^53^. This contrasts with GlcA, which strongly prefers the ^4^C_1_ conformation^54^. In the IdoA co-crystal structure, the ^4^C_1_ conformation is stabilized by a salt bridge between the IdoA carboxylate group and the side chain of R189. This stabilization is lost in the R189A mutation, leading to a preferential impairment of the activity HS2ST toward IdoA-containing substrates, relative to substrates containing GlcA^27^. Upon 2-O-sulfation, the ^4^C_1_ conformation of IdoA is further destabilized^53,55^, which we hypothesize leads to a disruption of its interaction with R189, ultimately enhancing the rate of product release. The higher activity of the wild type enzyme for IdoA, relative to GlcA, is then explained by the fact that both GlcA and GlcA2S are stable in the ^4^C_1_ conformation^54^, and thus would be predicted to be released from the enzyme more slowly.

## Methods

### Non-Glyc-HS2ST expression, purification, and mutagenesis

The chicken HS2ST-Maltose Binding Protein (MBP) fusion protein was expressed and purified according to previous studies^27^. The fusion protein was transformed into *E*. *coli* Origami B (DE3) cells, cultured at 37 °C until OD_600_ reached 0.5, then induced with isopropyl-β-D-thiogalactopyranoside and allowed to grow overnight at 20 °C. Cells were sonicated, followed by a two-step purification procedure. Whole-cell supernatant was passed through an amylose column with Tris buffer (25 mM Tris, 500 mM NaCl, pH 7.5). An elution buffer consisted of Tris Buffer with 40 mM Maltose. The amylose column eluent was concentrated and loaded on a Superdex 200 Increase 10/300 GL with PBS buffer (10 mM sodium phosphate, 150 mM NaCl, pH 7.8) to separate trimeric protein from aggregate and independent MBP (Supplementary Fig. 4). All reported mutations formed homotrimeric complexes according to the SEC profiles, including the negative control (NC = R189A/R80E/R184E). Protein concentration was assessed via UV at 280 nm (MW of trimer ~210 KDa, extinction coefficient ~119,000 M^−1^ cm^−1^ according to ExPASy ProtParam webtool^56^). The trimeric protein preparation was adjusted to 5% glycerol and stored at −20 °C until use. Additional non-glyc-HS2ST constructs were created with the Q5® Mutagenesis kit (New England Biolabs Inc.; cat. # E0554S) and sequenced at the Georgia Genomics Facility at the University of Georgia.

### Glyc-HS2ST expression and purification

An expression construct was generated encoding the truncated catalytic domain of human HS2ST as an NH_2_-terminal fusion protein in the pGEn2 expression vector essentially as described in prior studies^57^. The fusion protein coding region was comprised of a 25-amino acid signal sequence, an His_8_ tag, AviTag, the “superfolder” GFP coding region, the 7-amino acid recognition sequence of the tobacco etch virus (TEV) protease followed by the human HS2ST catalytic domain region comprising of 328 amino acid residues. The recombinant human HS2ST was expressed as a soluble secreted protein by transient transfection of suspension culture HEK293-F cells (FreeStyle^TM^ 293-Fcells, Thermo Fisher Scientific, Waltham MA) maintained at 0.5–3.0 × 10^6^ cells/ml in a humidified CO_2_ platform shaker incubator at 37 °C with 55% humidity. Transient transfection was performed using HEK293-F cells in the expression medium comprised of a 9:1 ratio of Freestyle^TM^293 expression medium (Thermo Fisher Scientific, Waltham MA) and EX-Cell expression medium including Glutmax (Sigma-Aldrich). Transfection was initiated by the addition of plasmid DNA and polyethyleneimine as transfection reagent (linear 25-kDa polyethyleneimine, Polysciences, Inc., Warrington, PA) as previously described^58,59^. Twenty-four hours post-transfection the cell cultures were diluted with an equal volume of fresh media supplemented with valproic acid (2.2 mM final concentration) and protein production was continued for an additional 4–5 days at 37 °C. The cell cultures were harvested, clarified by sequential centrifugation at 1200 rpm for 10 min and 3500 rpm for 15 min at 4 °C, and passed through a 0.8 µM filter (Millipore, Billerica, MA). The enzyme preparation was adjusted to contain 20 mM HEPES, 20 mM imidazole, 300 mM NaCl, pH 7.5, and subjected to Ni^2+^-NTA Superflow (Qiagen, Valencia, CA) chromatography using a column prequilibrated with 20 mM HEPES, 300 mMNaCl, 20 mM imidazole, pH 7.5 (Buffer I). Following loading of the sample the column was washed with 3 column volumes of Buffer I followed by 3 column volumes of Buffer I containing 50 mM imidazole, and eluted with Buffer I containing 300 mM imidazole at pH 7.0. The protein was concentrated to approximately 3 mg/ml using an ultrafiltration pressure cell (Millipore, Billerica, MA) with a 10-kDa molecular mass cutoff membrane. The NH_2_-terminal GFP fusion sequences were removed by *in vitro* cleavage with TEV protease, which cleaves at the boundary of the HS2ST and the fusion sequences. His/GFP-tagged TEV protease^60,61^ expressed in *E*. *coli* was added to the concentrated protein sample in the Ni^2+^-NTA elution buffer at a ratio of 1:5 relative to the fusion protein and incubated at 4 °C for 24 h. The cleaved HS2ST was further separated from the fusion tag and protease by Ni^2+^-NTA chromatography and gel filtration on a Superdex G-75 column (GE Healthcare) preconditioned with a buffer containing 20 mM HEPES, 100 mM NaCl, 0.05% Sodium azide, pH 7.0. Peak fractions of HS2ST were collected, buffer-exchanged into PBS and adjusted to 5% glycerol (Supplementary Fig. 5). The final protein preparation was stored at −20 °C until use. Protein concentration was determined via UV at 280 nm (MW of trimer ~117 KDa, extinction coefficient ~53,000 M^−1^ cm^−1^ as predicted by the ExPASy ProtParam webtool^56^).

### Bio-layer interferometry (BLI)

The Octet® RED96 system (FortéBio Inc.) was employed to detect the affinity of the enzymes for specific HS targets. Due to the small size of the glycan and the deep HS2ST binding cavity, the glycan and protein were treated as ligand and analyte, respectively. Varyingly sulfated HS fragments conjugated to biotin linkers were purchased from Glycan Therapeutics Inc. with purities greater than 93% according to HPLC (sequences located in Supplementary Table 1). The glycans were immobilized onto streptavidin (SA) labeled biosensors (FortéBio; cat. #18–5019) at 0.5 µg/mL. Experiments were performed at 25 °C in PBS Buffer (10 mM sodium phosphate, 150 mM NaCl, pH 7.8) with a final concentration of 1X Pall Kinetics buffer (FortéBio Inc; cat. #18–1092). Fresh biosensors and protein samples were used for each measurement (no regeneration step).

#### Multi-sensor protocol

The kinetics protocol began with a custom step (120 sec), followed by glycan loading (720 sec). A baseline (120 sec) was then established before the association and dissociation steps (420 sec each).

#### Single-cycle protocol

Another BLI assay format was employed to reduce the number of biosensors required to collect maximum response values for the creation of equilibrium binding curves. In this format, the same sensor is exposed to increasing concentrations of the protein without a dissociation step (Supplementary Fig. 6). Each step matched the timescale described for the multi-sensor protocol.

#### Data analysis

Results were processed with the Octet Data Analysis software, version 9.0. A reference sensor consisting of a glycan-coated biosensor and blank association wells (PBS-only) was included in each experiment and subtracted from the maximum response values. The non-specific binding signals between the biosensors and HS2ST were low for the 500 nM range of protein concentrations (Supplementary Fig. 7a); however, parallel reference sensors were implemented for the high concentration range (2 µM) employed with the glyc-HS2ST and compound **2**. Equilibrium binding curves and affinity determinations were completed with Graphpad Prism V7.0 (Graphpad Prism Software Inc.) according to the one-site specific binding equation: Y = Bmax ∗ X/(Kd + X).

### Inhibition assay

The same glycan sequences were synthesized^62^ for the inhibition assay as were used for the binding studies (Supplementary Table 1), except these terminated in p-nitrophenol rather than the PEG4-Biotin linker. The reaction mixture contained Tris pH 7.5 50 mM, 0.3 µg non-glyc-HS2ST WT, 0.08 nmol ^35^S-labeled 3′-phosphoadenosine 5′-phosphosulfate ([^35^S] PAPS) (3 × 10^4^ cpm), completely de-*O*-sulfated *N*-sulfated heparin (CDNS heparin) 1 µg, and either compound **2**, **3**, **4** (0, 1, 2, 4, 8, and 16 µg) or **5** (2, 20, 60, and 160 µg) in a total volume of 0.1 ml. After incubation at 37 °C for 1 h, the reaction was quenched by adding 1 mL of 0.01% Triton X-100 buffer at pH 5.0 containing 150 mM NaCl, 50 mM NaOAc, 3 M urea and 1 mM ethylenediaminetetraacetic acid (EDTA). The samples were loaded on a small DEAE column (200 µl in size). The column was then washed five times with the same buffer, each time 1 ml. The column was further washed five times with 0.25 M NaCl in 0.001% Triton X-100 buffer each time 1 ml. The column was eluted with 1 ml of 1 M NaCl in 0.001% Triton X-100 buffer. The ^35^S cpm of the eluted samples were tested the by a scintillation counter.

### Flow cytometry

#### Chinese hamster ovary (CHO) cells

Wildtype CHO-K1, pgsD-677 (*Ext1*^−/−^), and pgs-F17 cells (*Hs2st*^−/−^)^43^ were grown as monolayers. Single cell suspensions were obtained by lifting the cells with Cell Dissociation Buffer (Life Technologies; cat. #13151014), sedimented by centrifugation, resuspended in ice cold PBS containing 0.1% BSA (Life Technologies; cat. # 14190250, Sigma; cat. # A3059–100G) and 3 × 105 cells were aliquoted to each well of a 96-well clear v-bottom plate (Fisher; cat. # 7200108). After centrifugation at 500 × g at 4 °C for 5 min, the cells were resuspended in 200 µL of in PBS/BSA containing 30 nM of non-glyc-HS2ST WT, mutants H142A, R189A, or the negative control (R189A/R80E/R184E). After 1 hr at 4 °C, the cells were centrifuged, washed twice with cold PBS/BSA, and resuspended in buffer containing 1 µg/ml of Maltose Binding Protein Monoclonal Antibody (R29) (Fisher; cat. # MA5–14122). After 1 hr at 4 °C, the cells were washed twice, resuspended in a solution containing Alexa Fluor 488-tagged goat anti-mouse IgG (1.25 µg/mL) (Fisher; cat. # A11001) for 30 min. The cells were washed with ice cold PBS and analyzed by flow cytometry (Benton Dickinson FACSCalibur cytometer). Data was analyzed using FlowJo and plotted with Graphpad Prism V7.0 (Graphpad Prism Software Inc.).

#### Mouse lung endothelial (MLE) cells

The *Hs2st*^−/−^ cell lines were generated by deriving immortalized primary MLE Cells from conditionally targeted (floxed) *HS2st* mice with SV40 large T-antigen and deleting the floxed gene by expressing Cre-recombinase. HS disaccharide composition analysis determined that the Cre-recombinase-mediated *Hs2st* deletion completely diminished 2-O-sulfation accompanied by increased N- and 6-O-sulfation (manuscript under review to report the generation and characterization of these two cell lines). Wildtype and *Hs2st*^−/−^ cells were cultured in DMEM high glucose medium supplemented with 10% FBS, penicillin (100 U/mL), and streptomycin (100 μg/mL). The cells were detached by PBS without Calcium and Magnesium supplemented with 2 mM EDTA and 0.5% BSA (PBS-E). Wildtype and mutant cells (5 × 105 cells) were re-suspended in 100 μL cold PBS-E and wildtype and mutant non-glyc-HS2ST protein were added to a final concentration of 1 μM. The cells were washed with PBS-E buffer three times after incubation on ice for 45 min and re-suspended in 100 μL cold PBS-E buffer. The anti-MBP antibody (MA5–14122, Invotrogen) was added to the cells (1:100) and incubated for 45 min. Following, the cells were washed three times with PBS-E buffer. The washed cells were incubated with Goat anti-mouse IgG secondary antibody conjugated with Alexa Fluor 488 (A11001, Invitrogen) for 45 min in 100 μL PBS-E buffer. The cells were washed with PBS-E buffer three times and re-suspended in 300 μl cold PBS-E buffer for flow cytometry (LSRII cytometer). Data was analyzed using Flowjo (Treestar) and plotted with Graphpad Prism V7.0 (Graphpad Prism Software Inc.).

### Molecular docking

Vina-Carb^45^, a modified version of Autodock Vina^63^, was used for docking. The starting structure of the HS-PAP-HS2ST complex was obtained from PDB ID: 4NDZ^33^. Heparan sulfate hexasaccharide structures were built with the GLYCAM Web tool (www.glycam.org). The resulting PDB files were processed via AutodockTools^64^. All glycosidic linkages, hydroxyl groups, and most sulfate torsions could rotate during the model generation procedure. The only explicitly restrained torsion angles were N–C, N–S, and O6–S. Ring conformations are not capable of transitioning by the program. The gridbox was centered on the catalytic histidine residue (H142), which corresponds to an x, y, and z coordinate of 2.465, 41.242, and 77.155, respectively. The dimensions of the x, y, and z lengths were set to 50, 40, and 20, respectively (Supplementary Fig. 8). The exhaustiveness was set to 80, 10x higher than the default, due to the number of rotatable bonds and size of the gridbox. The chi_coefficient and chi_cutoff were set to 1 and 2, respectively. RMSD values for docked structures were computed for the ring atoms of the acceptor and flanking residues, as depicted in Fig. 4a, with Chimera^65^.

### Molecular dynamics (MD) simulations

Chimera was employed to create a co-complex of the trimer bound to three ligands by aligning the docked model (with protein) to each of the remaining binding sites. MD simulations were performed with the pmemd.cuda version of AMBER14^66,67^. Amino acid and carbohydrate residues were parameterized with the FF12SB and GLYCAM06 (J-1) force fields, respectively^67,68^. The systems were neutralized and solvated with TIP3P in a truncated octahedral box with 15 Å distance from solute to the unit cell boundary. The Particle-Mesh Ewald algorithm accounted for electrostatic interactions^69^ and the non-bonded cutoff was set to 8 Å. SHAKE was employed to permit an integration time step of 2 fs. Restraints were imposed in specific situations and were enforced with a 10 kcal/mol Å^2^ energy barrier in each case. Three minimization steps were performed, each consisting of 1000 cycles of the steepest descent and 24,000 cycles of the conjugate gradient methods. The first restrained all solute atoms, the second restrained the protein backbone (Cα) and carbohydrate ring atoms (C1, C2, C3, C4, C5, O5), and the last was completely unrestrained. The systems were then heated to 300 K under NVT conditions over 60 ps, employing the Berendsen thermostat with a 1 ps coupling constant. The subsequent simulations were performed under NPT conditions for 200 ns.

### Computational analysis

Analysis was performed on the latter 50 ns of the 200 ns simulations. Root mean square fluctuations (RMSF) were generated using the cpptraj module of AmberTools14^70^. Molecular Mechanics-Generalized Born Surface Area (MM-GBSA) calculations were employed to quantify the total ΔG_bind_ between the HS2ST receptor and HS ligand^71,72^. The igb values and internal dielectric (idel) constants were set to 2 and 4, respectively, according to a previous study of sulfated ligands^73^. Images were created using the Visual Molecular Dynamics (VMD) program^74^. Cartoon representations of glycans follow the SNFG format^75^, and the 3D shapes were created with the 3D-SNFG plugin for VMD^76^.

## Electronic supplementary material

Supplementary Material
