# Author Correction: Downstream Products are Potent Inhibitors of the Heparan Sulfate 2-O-Sulfotransferase

**DOI:** 10.1038/s41598-020-66543-3

**Published:** 2020-06-09

**Authors:** David F. Thieker, Yongmei Xu, Digantkumar Chapla, Chelsea Nora, Hong Qiu, Thomas Felix, Lianchun Wang, Kelley W. Moremen, Jian Liu, Jeffrey D. Esko, Robert J. Woods

**Affiliations:** 1 Department of Biochemistry and Molecular Biology, University of Georgia, Athens, GA 30602 USA; 2 Complex Carbohydrate Research Center, University of Georgia, Athens, GA 30602 USA; 3 Division of Chemical Biology and Medicinal Chemistry, Eshelman School of Pharmacy, University of North Carolina, Rm 1044, Genetic Medicine Building, Chapel Hill, USA; 4 Department of Cellular and Molecular Medicine, University of California San Diego, La Jolla, California USA

Correction to: *Scientific Reports* 10.1038/s41598-018-29602-4, published online 07 August 2018

In this Article, the authors neglected to include some potential conflicts of interest.

The Competing Interests section should read:

“J. L. is a founder of Glycan Therapeutics. D.F.T., Y.X., D.K., C.N., T.F., H.Q., L.W., K.W.M., R.J.W. declare no competing interests. J.D.E. is a co-founder of TEGA Therapeutics. J.D.E. and The Regents of the University of California have licensed a University invention to and have an equity interest in TEGA Therapeutics. The terms of this arrangement have been reviewed and approved by the University of California, San Diego in accordance with its conflict of interest policies.”

