# Supplementary material for: Downstream Products are Potent Inhibitors of the Heparan Sulfate 2-O-Sulfotransferase
Source: Sci Rep. 2018 Aug 7;8:11832. doi: 10.1038/s41598-018-29602-4 (PMC6081452; doi:10.1038/s41598-018-29602-4)
Supplement: Supplementary file 1 — Supplementary Material [file 41598_2018_29602_MOESM1_ESM.docx]

**Supplementary Material**

**Downstream Products are Potent Inhibitors of the Heparan Sulfate 2-O-Sulfotransferase**

David F. Thieker^1,2^, Yongmei Xu^3^, Digantkumar Chapla^2^, Chelsea Nora^4^, Hong Qiu^2^, Thomas Felix^1,2^, Lianchun Wang^1,2^, Kelley W. Moremen^1,2^, Jian Liu^3^, Jeffrey D. Esko^4^, and *Robert J. Woods^1,2^

*^1^Department of Biochemistry and Molecular Biology, University of Georgia, Athens, GA 30602 USA; ^2^Complex Carbohydrate Research Center, University of Georgia, Athens, GA 30602 USA; ^3^Division of Chemical Biology and Medicinal Chemistry, Eshelman School of Pharmacy, University of North Carolina, Rm 1044, Genetic Medicine Building, Chapel Hill, USA; ^4^Department of Cellular and Molecular Medicine, University of California San Diego, La Jolla, California, USA*

*To whom correspondence should be addressed: e-mail: rwoods@ccrc.uga.edu

**Supplementary Table 1. Oligosaccharide sequences for HS compounds.**

| **Compound** | **Sulfation** | **Glycan Sequence** | **Charge** |
| --- | --- | --- | --- |
| **1** | NS | GlcNS-GlcA-GlcNS-IdoA-GlcNS-GlcA~ | -6 |
| **2** | NS/2S | GlcNS-GlcA-GlcNS-IdoA2S-GlcNS-GlcA~ | -7 |
| **5** | NS/6S | GlcNS6S-GlcA-GlcNS6S-GlcA-GlcNS6S-GlcA~ | -9 |
| **3** | NS/2S/6S | GlcNS6S-GlcA-GlcNS6S-IdoA2S-GlcNS6S-GlcA~ | -10 |
| **4** | NS/2S/6S/3S | GlcNS6S-GlcA-GlcNS3S6S-IdoA2S-GlcNS6S-GlcA~ | -11 |

^a^”~” indicates the biotinylated linker, see Supplementary Fig. 9 for complete structure

**
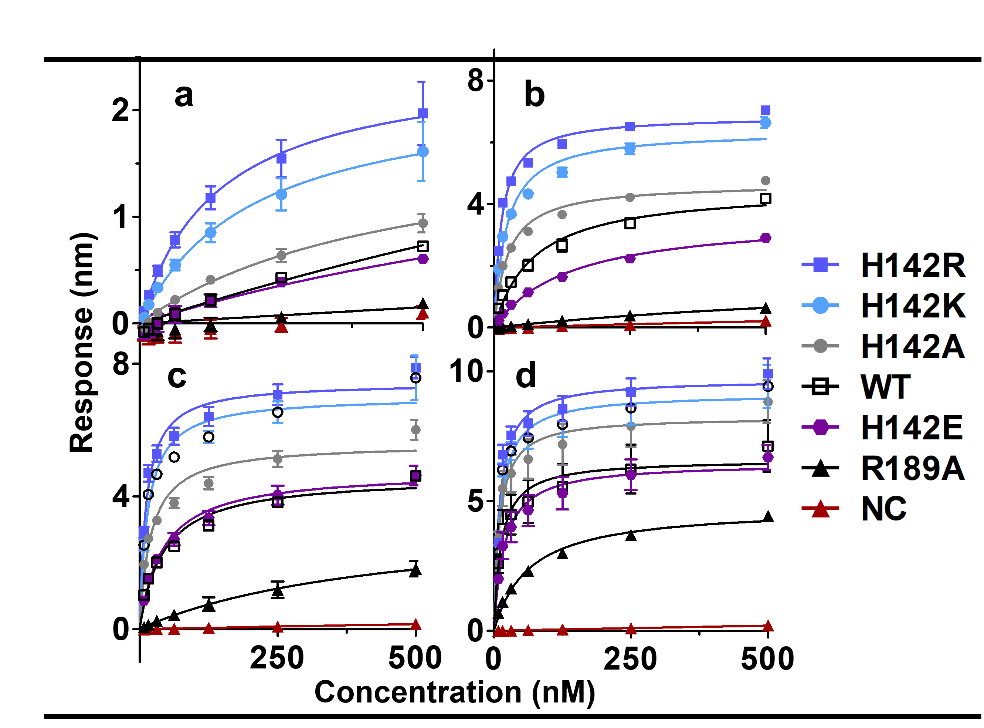
**

**Supplementary Figure 1. Effects of mutating putative non-glyc-HS2ST binding site residues. (a-d)** Saturation binding curves (n=3) that correspond with Table 3 are depicted for **2** (b), **5** (c), **3** (d), and **4** (e). Binding responses for **1** were insignificant (Supplementary Fig. 1).

**
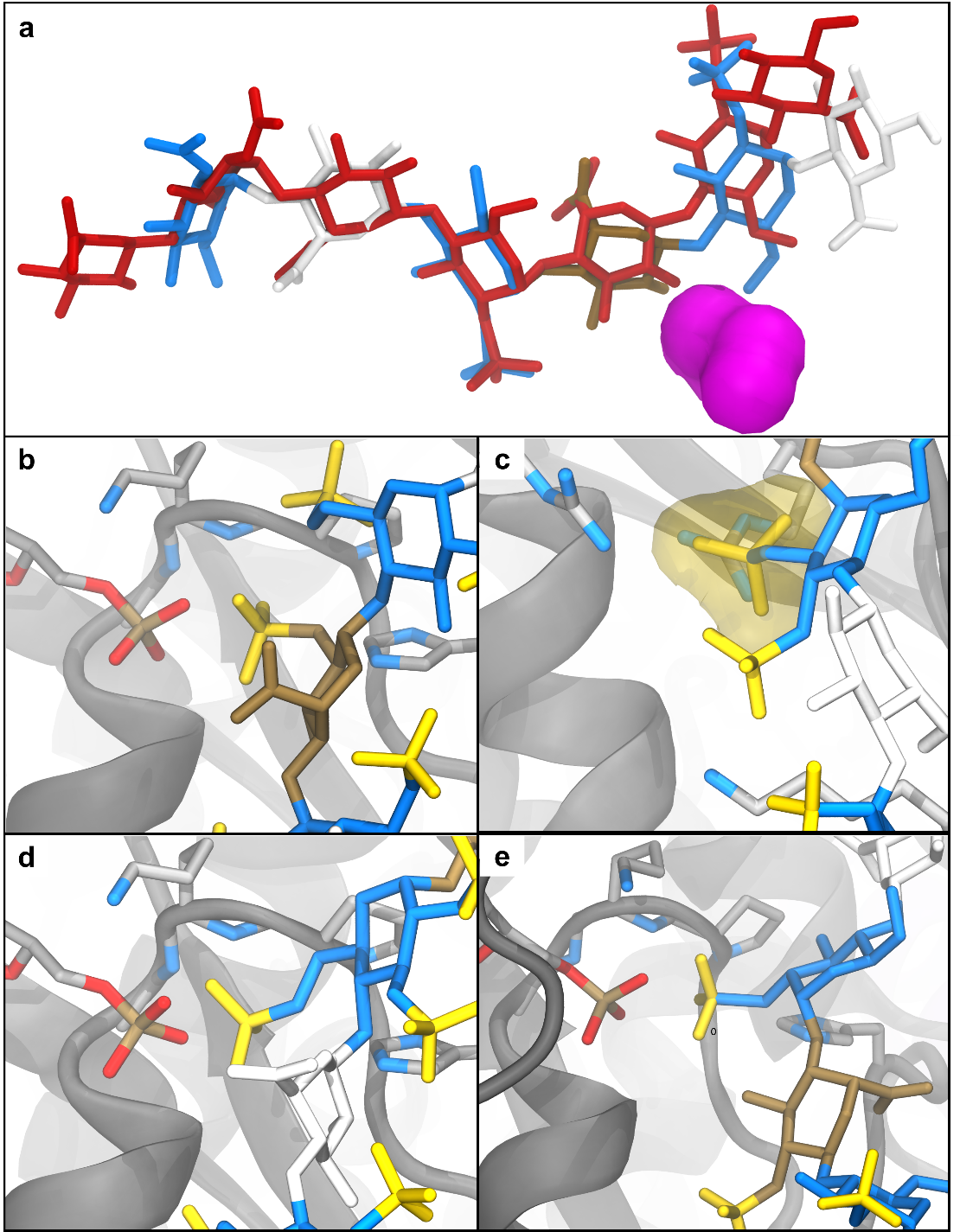
**

**Supplementary Figure 2. Docked models for the HS2ST. (a)** The top-ranked structure from docking **1** (colored according to residue name) is superimposed on top of the structure of the co-complex with bound heptasaccharide (red). A surface representation of the catalytic H142 residue is also displayed for context (magenta). **(b, c, d, e)** PAP is displayed for context (faded gray, left), but was not present during the docking procedure. The catalytic residue (H142) and PSB loop are also depicted. **(b)** Model of compound **3** in which the 2-sulfate fits in the PSB loop. **(c)** Model of **4** highlighting the inability for the NS (yellow surface) to fit in the N-sulfate binding site due to the attached 3S moiety. This image is comparable to Fig. 6c. **(d)** A different model of **4** in which the 6S is positioned in the PSB loop. **(e)** Model of **2** in which the chain proceeds in the reverse direction compared to the substrate within the crystal structure.

**
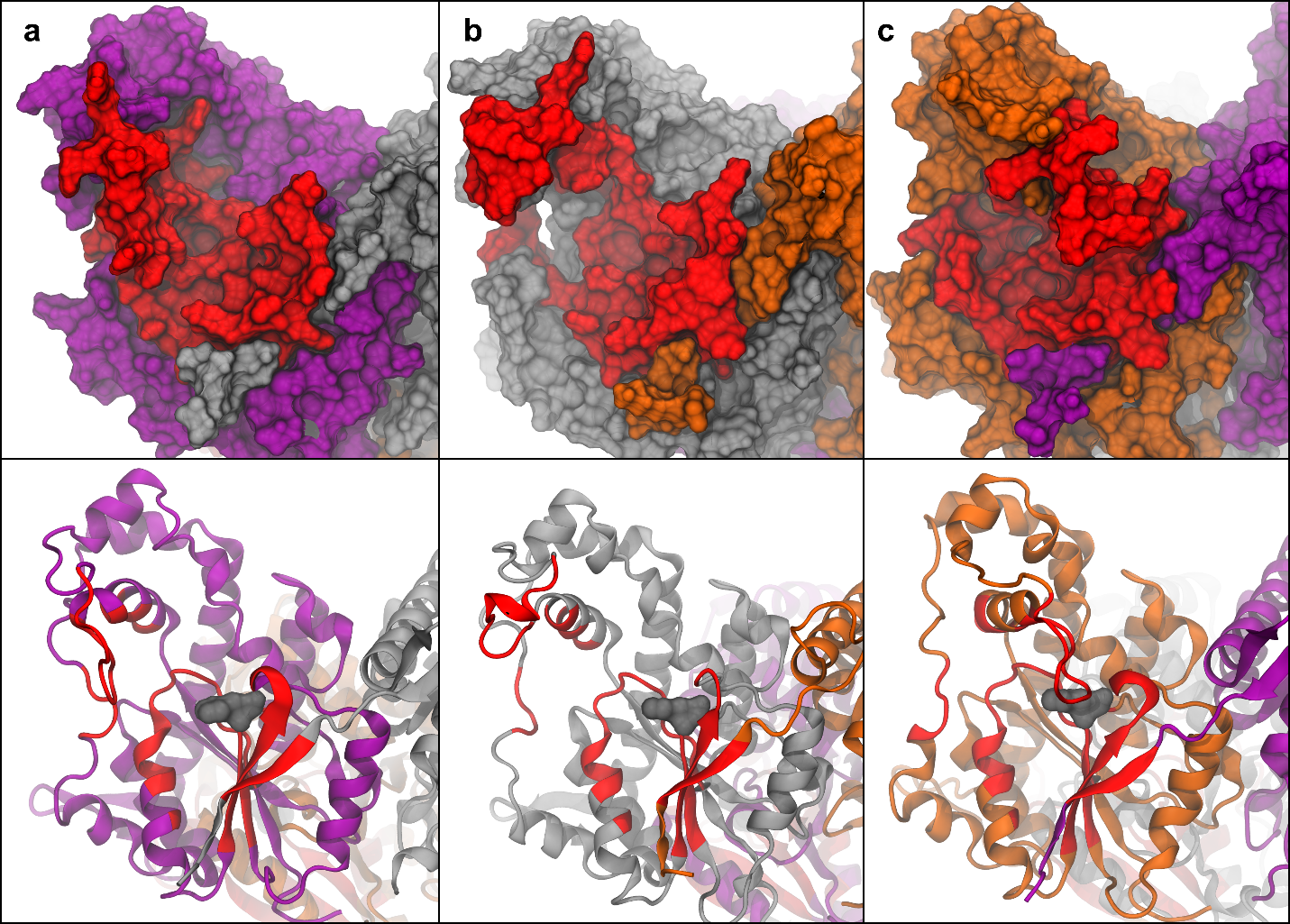
**

**Supplementary Figure 3. Mobility of active site during MD.** **(a, b, c)** A 200 ns simulation of the apo-protein yielded varying configurations of the active site in each monomer of the trimeric protein. Each monomer is colored differently, and amino acids within 10 Å of the acceptor IdoA from the co-complex (PDB ID: 4NDZ) are highlighted in red. **(a)** The first active site is similar to the crystal structure. **(b)** The second active site expanded. **(c)** The third active site demonstrated a closed HS-binding site due to a loop that extended to cover the catalytic residue.

**
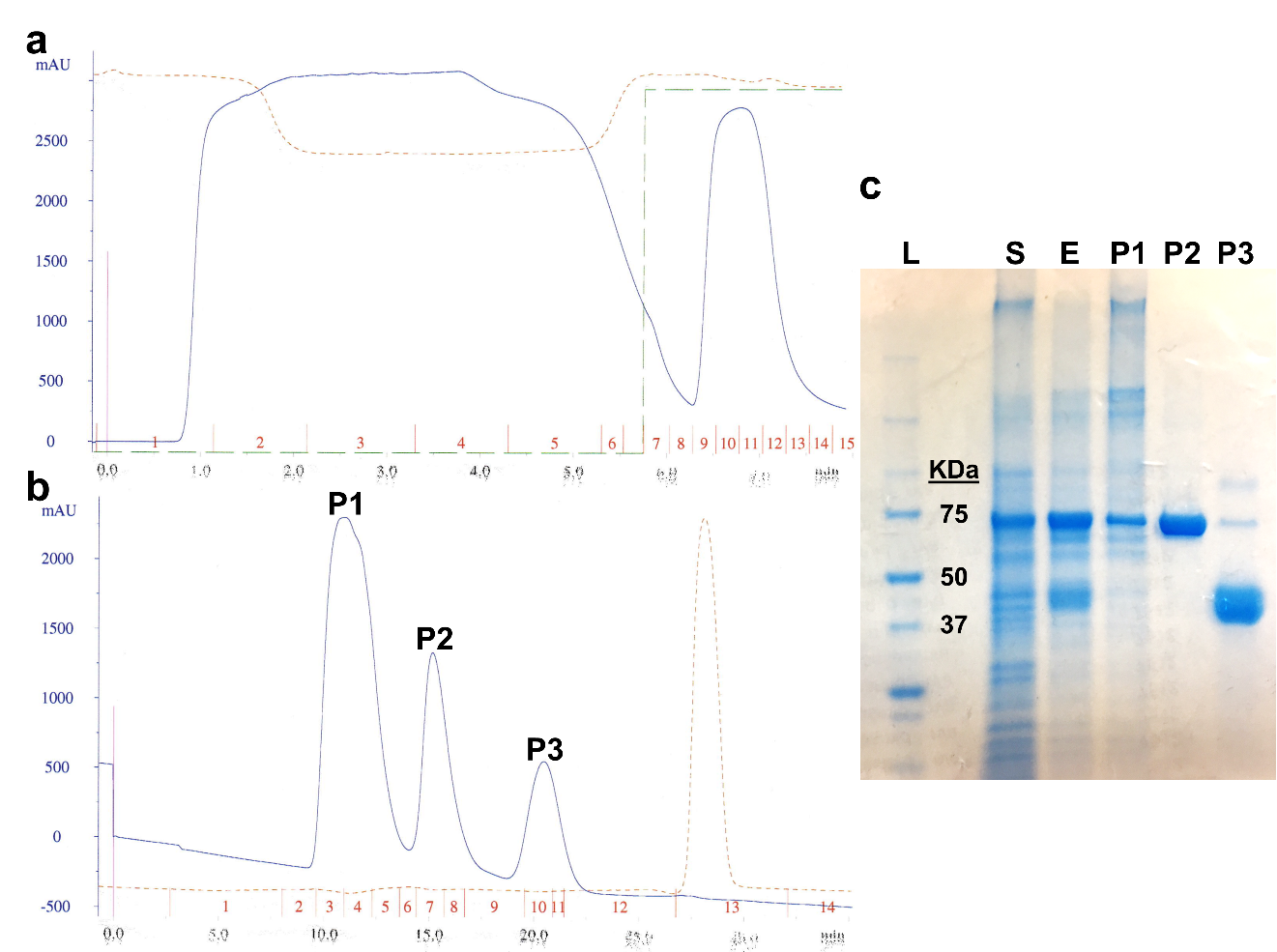
**

**Supplementary Figure 4. Purification procedure for non-glyc-HS2ST.** **(a)** A representative chromatogram from loading the supernatant (S) of the WT cell lysate on an Amylose column. The UV trace (blue), conductivity (brown dashed), fraction number (red), and time (black) are depicted. Fractions 10-12 were collected as the eluent (E). **(b)** A representative chromatogram from separating E with a size exclusion column. The three peaks (P1-3) are labeled according to the elution order. **(c)** A SDS-PAGE that includes samples from (a) and (b). Three bands of the protein standards (L) are annotated with respective molecular masses. P1 is a protein aggregate, P2 contains the trimeric non-glyc-HS2ST protein, and P3 is predominantly free Maltose Binding Protein (MBP).

**
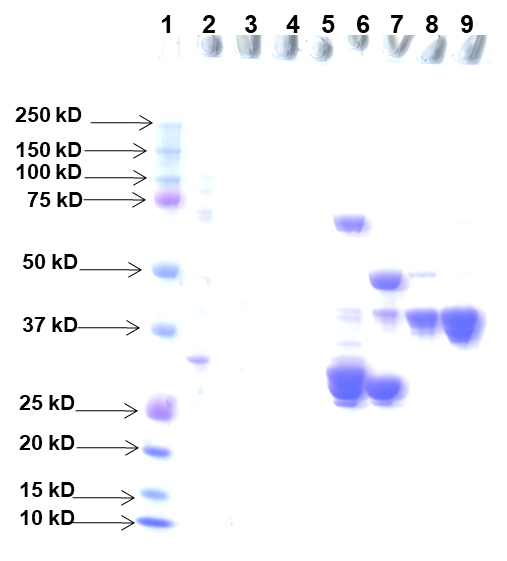
**

**Supplementary Figure 5. Strategy for glyc-HS2ST expression, purification, and tag cleavage.** Lanes, 1: Molecular weight marker; 2: HS2ST in crude media; 3: Flow through Ni-NTA; 4: Ni-NTA wash with Buffer I; 5: Ni-NTA wash with Buffer II; 6: Ni-NTA elution of HS2ST; 7: HS2ST+TEV for Tag cleavage; 8: HS2ST from run through Ni-NTA 2; 9: Pooled HS2ST from Gel filtration.

**
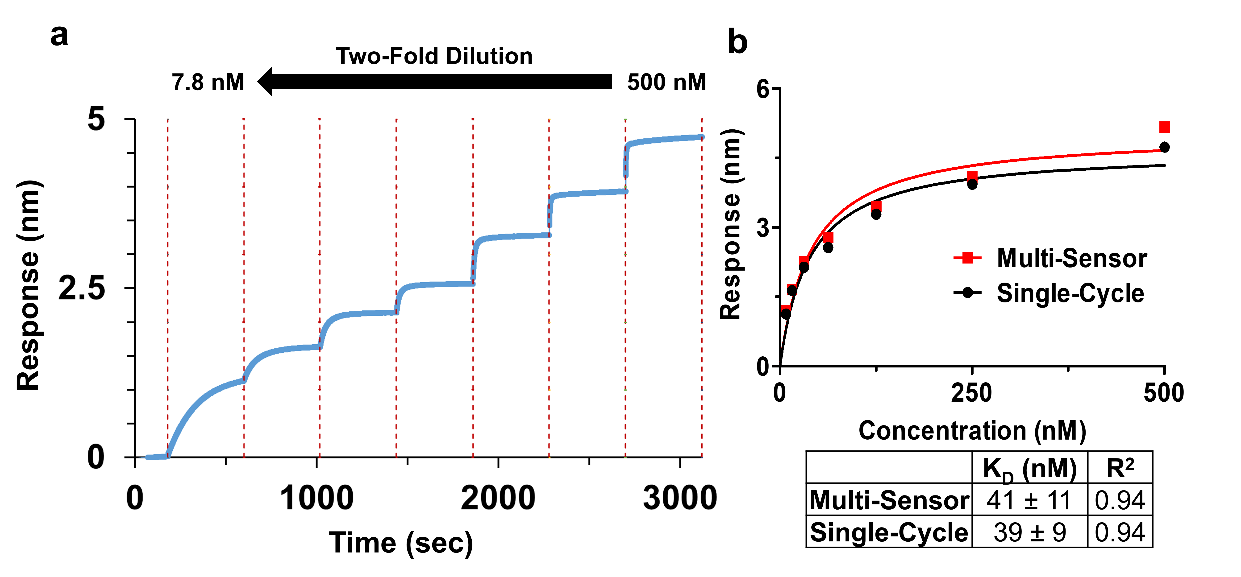
**

**Supplementary Figure 6. BLI assays.** **(a)** Example sensorgram from a single-cycle experiment consisting of non-glyc-HS2ST WT in a two-fold dilution series (7.8 – 500 nM) with compound **3**. **(b)** Comparison of saturation binding curves derived between the multi-sensor assay and single-cycle assay formats (n=1). The same protein sample was used for both assays.

**
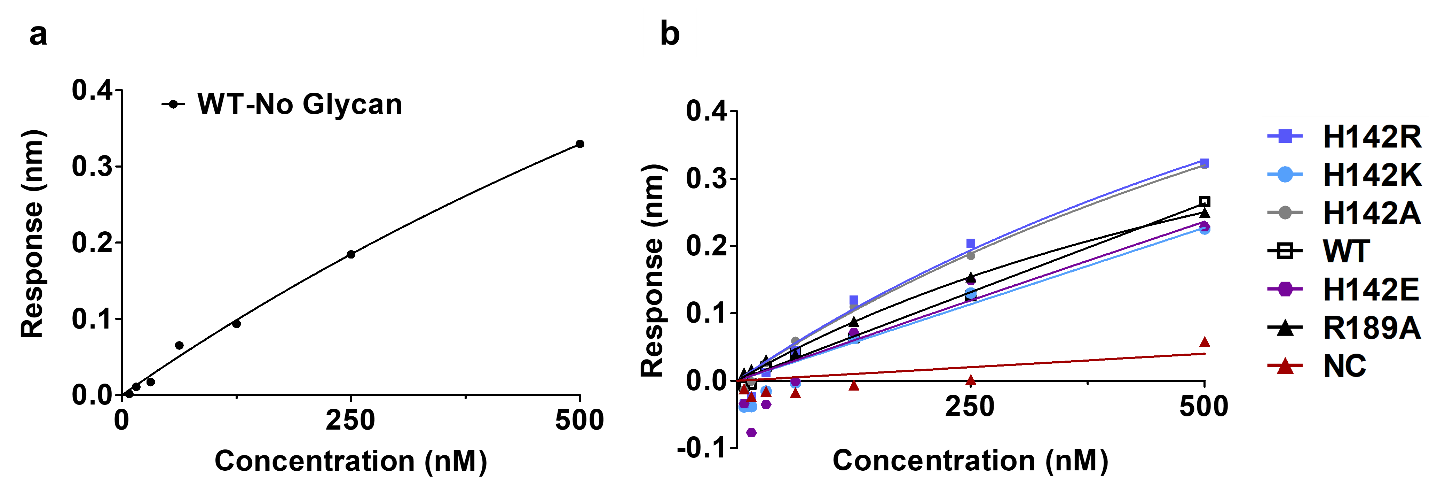
**

**Supplementary Figure 7. Non-glyc-HS2ST saturation binding profiles.** **(a)** Binding response (n=1) between the WT enzyme and the streptavidin-coated surface (no glycan loading step) indicate non-specific binding signals. **(b)** Saturation binding curves (n=1) for **1**. These responses match the non-specific binding responses of (a) and therefore are insufficient for K_D_ determination.

**
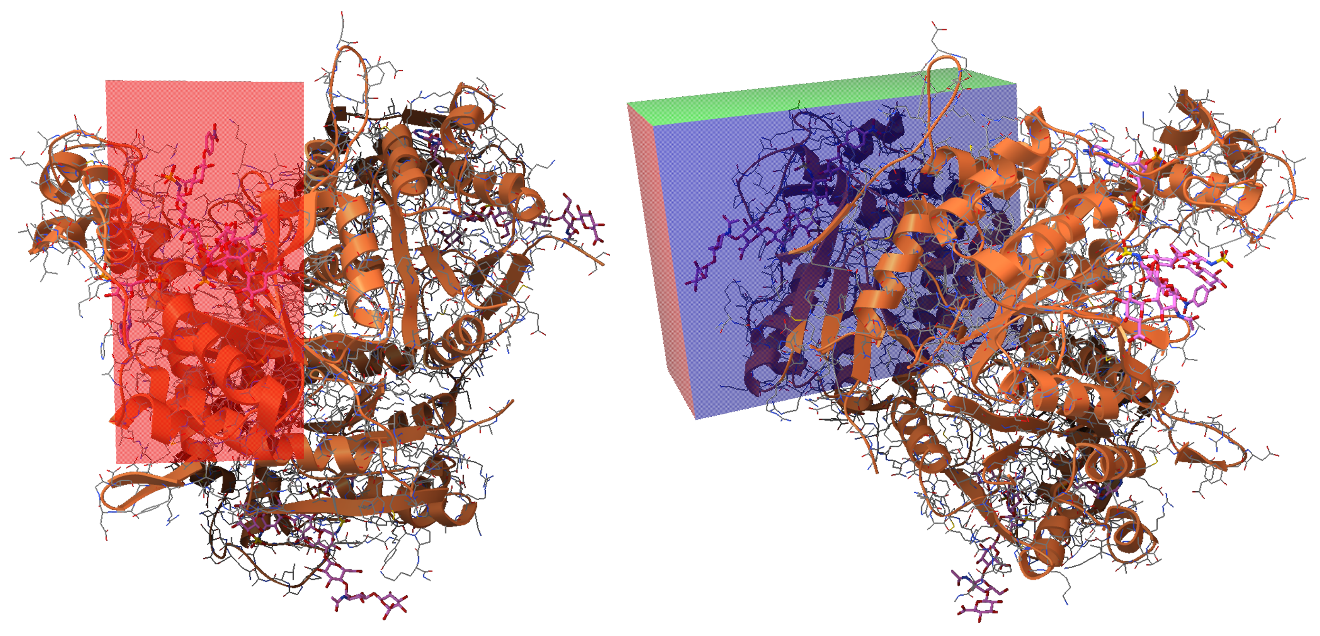
**

**Supplementary Figure 8. Autodock grid box.** Two orientations of the docking grid box are depicted with the HS2ST co-crystal structure (PDB ID: 4NDZ), one from the bottom (left) and the other from the side (right). The trimeric protein is depicted with an orange cartoon and ligands are represented in pink.

**
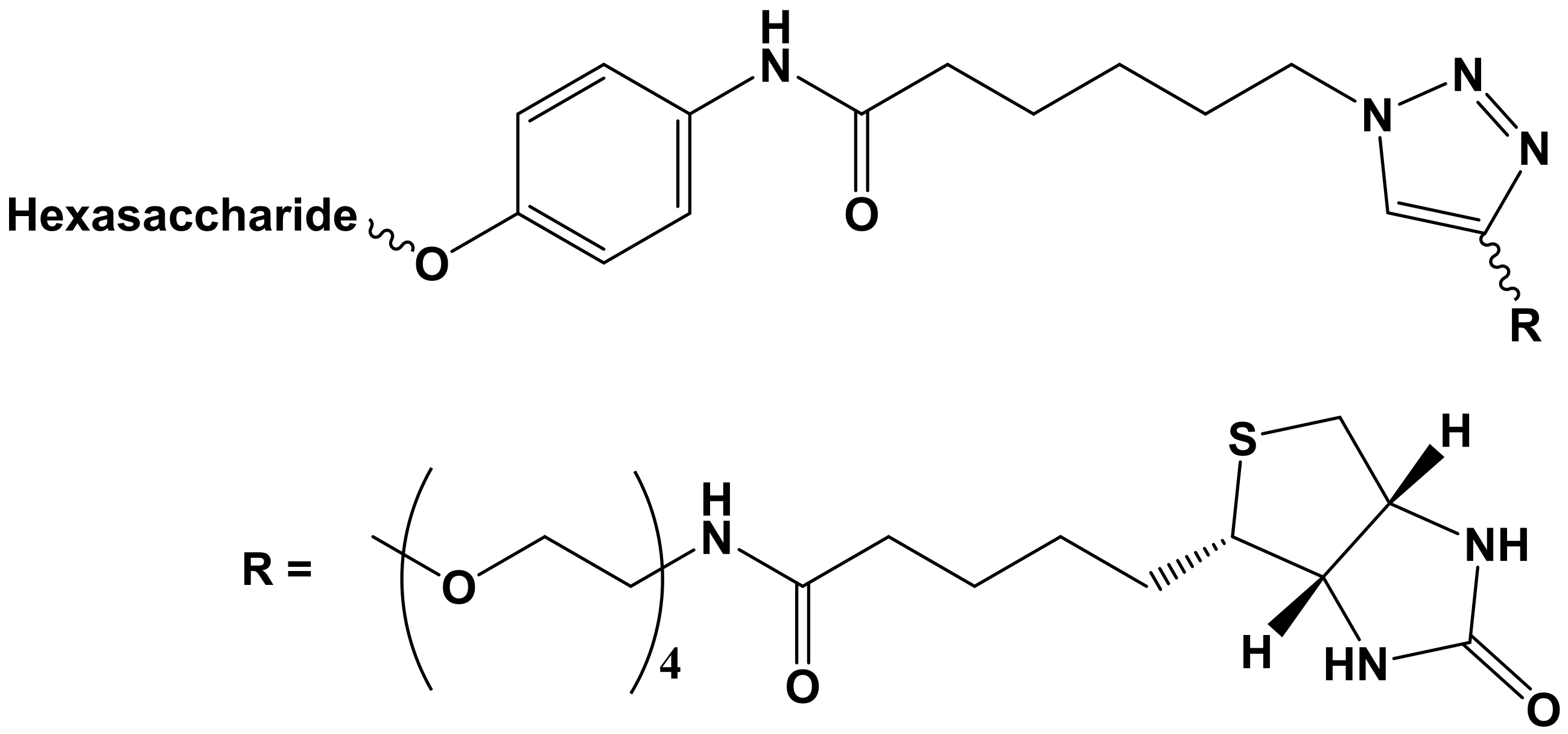
**

**Supplementary Figure 9. Structure of glycan linker.** Hexasaccharide ligands described in Supplementary Table 1 were linked to biotin with azide-alkyne click polymerization. A polyethylene glycol repeat (4x) acts as a spacer between the two units.
